# Financial Literacy and Financial Toxicity Among US Veterans: Cross-Sectional Survey Informing Public Health Informatics Screening

**DOI:** 10.2196/97291

**Published:** 2026-08-07

**Authors:** Harman Sabharwal, Ramona Raya

**Affiliations:** 1 Langley High School, 6520 Georgetown PikeMcLean, VA, 22101, United States, +1703-459-3559; 2 Department of Medical Education, University of Virginia, Charlottesville, VA, United States

**Keywords:** financial toxicity, veterans, public health informatics, screening, digital health, health equity, social determinants of health, risk stratification, benefit navigation

## Abstract

**Background:**

Financial toxicity can contribute to adverse health and care-access outcomes among US veterans, yet scalable methods to identify individuals at elevated risk remain limited. Public health informatics frameworks may enable the translation of patient-reported financial risk signals into streamlined screening, risk stratification, and care-navigation workflows.

**Objective:**

This study aimed to examine concept-level indicators of financial literacy and financial toxicity among US veterans and explore how these findings could inform future informatics-enabled screening strategies for identifying subgroups at increased risk of health-related financial strain.

**Methods:**

We conducted an exploratory cross-sectional survey of 88 US veterans from 2024 to 2025. Financial literacy was assessed using 3 benchmark items from the National Financial Capability Study. Financial toxicity was assessed using items aligned with domains reflected in the Comprehensive Score for Financial Toxicity framework, including difficulty affording care, reduced or quit work, borrowing money or using savings for care, and treatment-adherence impact. Analyses included descriptive statistics, Fisher exact tests, unadjusted logistic regression, and a minimally adjusted sensitivity model for work disruption, controlling for age and education.

**Results:**

Female veterans had lower rates of high financial literacy than male veterans (15/29, 52% vs 48/59, 81%; *P*=.006) and lower correct-response rates on compound interest (10/29, 35% vs 36/59, 61%; *P*=.02) and inflation (14/29, 48% vs 43/59, 73%; *P*=.03). Black veterans had lower correct-response rates than non-Black veterans on inflation (11/24, 46% vs 46/64, 72%; *P*=.03) and retirement strategy (15/24, 63% vs 56/64, 88%; *P*=.014), although composite high-literacy rates did not differ significantly by race. In unadjusted models among participants with complete outcome data (n=75), lower financial literacy was directionally associated with higher odds of all 4 financial toxicity outcomes, with the clearest association observed for work disruption (odds ratio 0.56 per 1-point increase in financial literacy score, 95% CI 0.33-0.95; *P*=.03). Black female veterans reported elevated financial toxicity across multiple domains. Financial support program use was low overall (29%).

**Conclusions:**

These findings suggest that financial literacy may be a marker of vulnerability to financial toxicity among veterans, but observed associations should be regarded as preliminary and hypothesis-generating. The results identify concept-level financial literacy domains and work disruption as candidate signals for future screening evaluation. Future research should evaluate whether brief screening, financial literacy assessment, and benefit-navigation strategies improve identification, referral, adherence, and downstream financial and health-related outcomes in larger and more representative veteran populations.

## Introduction

Financial toxicity, defined as the financial strain arising from health care costs and its downstream material, psychological, and behavioral consequences, was initially recognized and operationalized in oncology research. Cancer care provided an early setting for this concept because patients often face prolonged treatment courses, high out-of-pocket expenses, insurance complexity, employment disruption, and difficult trade-offs between treatment adherence and household financial stability [1-3]. Early oncology work framed financial toxicity as a patient-reported outcome because financial strain could affect not only material well-being but also quality of life, symptom burden, treatment decisions, and adherence [2,3]. Although the construct originated in cancer care, similar mechanisms may operate in other populations facing chronic illness, fragmented benefits, employment disruption, and health care affordability barriers.

Among US veterans, financial well-being is closely linked to post-service health, housing stability, and reintegration into civilian life [4-7]. Prior research has documented substantial heterogeneity in financial outcomes within veteran populations, with disparities across race, sex, and socioeconomic status [8,9]. Female veterans face distinct structural and economic challenges, including lower earnings and higher unmet health care needs compared with male veterans [10,11]. Veterans of color are also more likely to experience housing instability and adverse social determinants of health, which may negatively affect both financial and health outcomes [12,13].

Financial literacy, defined as the ability to understand and apply core economic concepts such as compounding, inflation, and investment diversification, plays an important role in financial decision-making and long-term economic resilience [14]. Lower financial literacy has been associated with a greater debt burden, poorer financial behaviors, and heightened vulnerability to financial shocks [15,16]. In health care settings, these vulnerabilities may exacerbate financial toxicity when individuals face income disruption, out-of-pocket medical expenses, or complex benefit systems. However, the relationship between specific financial literacy concept deficits and health-related financial toxicity has not been empirically examined in veteran populations. In this study, financial literacy refers specifically to 3 benchmark financial concepts assessed in the National Financial Capability Study (NFCS)—compound interest, inflation, and retirement diversification—rather than broader financial capability or health-insurance literacy.

Prior work has documented racial and sex-based differences in financial capability among US veterans using large national samples [8,9]. These studies showed that female veterans and veterans of color reported lower financial capability scores than their White male counterparts. However, prior analyses relied largely on composite financial capability measures and did not assess whether lower performance on specific financial literacy concepts was associated with health-related financial toxicity outcomes such as treatment-adherence impact, work disruption, or borrowing for care. As a result, it remains unclear whether financial literacy gaps and financial toxicity burdens reflect similar or different patterns across veteran subgroups. Whether observed subgroup differences in literacy reflect independent racial and gender effects or are partially attributable to differences in educational attainment or age composition within this convenience sample warrants careful interpretation. A concept-level approach may provide more actionable insight by identifying which domains of financial knowledge appear most limited among veterans. Emerging public health frameworks suggest that examining race and sex independently may obscure compounded vulnerabilities experienced by specific subgroups [17,18]. Therefore, exploratory subgroup analysis may help identify patterns that warrant further study in larger and more representative veteran samples.

This study examines concept-level financial literacy and financial toxicity indicators among US veterans and explores preliminary associations between financial literacy and health-related financial strain. We examine subgroup patterns by race and self-reported sex, including an exploratory analysis of the Black female subgroup, descriptively to identify areas for future research rather than to draw confirmatory conclusions. We hypothesize that financial literacy differs across racial and sex subgroups and that lower financial literacy is associated with higher financial toxicity. Beyond describing exploratory subgroup patterns, this study identifies candidate indicators that could inform future evaluations of public health informatics approaches for brief financial toxicity screening, risk stratification, and benefit-navigation support for veterans facing health care affordability barriers.

## Methods

### Research Objectives

The objectives of this study were to (1) describe financial toxicity indicators among US veterans; (2) evaluate concept-level financial literacy across subgroups defined by race and sex; and (3) explore preliminary associations between financial literacy and financial toxicity, with a minimally adjusted sensitivity analysis for work disruption controlling for age and education.

### Study Design and Population

This cross-sectional survey examined financial literacy and financial toxicity among US veterans. Eligible participants were adults aged 18 years or older who self-identified as US veterans. Participants were recruited between July 2024 and April 2025 through an anonymous survey link distributed via veteran-focused community contacts, online veteran networks, and in-person outreach at local community events. No financial incentive was provided. Because recruitment was anonymous and community-based, the number of individuals who received or viewed the survey invitation could not be determined. In-person outreach occurred primarily in the Northern Virginia or Washington, DC, metropolitan region, while online recruitment may have reached veterans outside this area. The survey was administered through an open, anonymous Google Forms link without log-in or collection of direct identifiers; duplicate-entry prevention measures, such as cookies or IP-address checks, were not used.

Individuals who were younger than 18 years and did not identify as veterans were excluded. Additionally, participants with incomplete responses to all 3 financial literacy items were also excluded from the financial literacy analyses. The final analytic sample for financial literacy analyses included 88 participants. Financial toxicity items were completed by 75 of the 88 participants; therefore, analyses involving financial toxicity outcomes were based on this subsample, with valid-response denominators reported as applicable to each outcome.

Participation was voluntary and anonymous. Reporting was guided by the STROBE (Strengthening the Reporting of Observational Studies in Epidemiology) statement [19]. Because this study was exploratory and based on a modest community sample, no formal a priori power calculation was performed. This was a nonprobability convenience sample and was not designed to be nationally representative of all US veterans. Analyses were intended to be descriptive and hypothesis-generating, with limited power for complex multivariable analyses and small subgroup inference.

### Measures

Financial literacy was assessed using 3 benchmark items adapted from the NFCS, covering compound interest, inflation, and retirement strategy. Each item was coded as correct or incorrect, with “don’t know” and nonresponse coded as incorrect. Correct responses were defined as: “More than US $1150” for compound interest, “Less” for inflation, and “Investing in a mix of stocks and bonds” for retirement strategy. Scores were summed to create a composite financial literacy score ranging from 0 to 3, with higher scores indicating greater financial literacy. For descriptive and logistic analyses, financial literacy was additionally dichotomized as low financial literacy (0‐1 correct) vs high financial literacy (2‐3 correct). These 3 items were selected because they draw on the core concepts assessed by the widely used “Big Three” financial literacy questions [14]—interest compounding, inflation, and risk diversification (here operationalized as retirement or investment diversification). The full NFCS instrument was not administered to reduce respondent burden in a brief community-based veteran survey and to focus on the concepts plausibly relevant to financial vulnerability and health-related financial strain. The resulting score should therefore be interpreted as a brief concept-level literacy measure rather than a comprehensive assessment of financial capability.

Financial toxicity was assessed using items adapted from and aligned with the domains reflected in the Comprehensive Score for Financial Toxicity (COST) framework, rather than the validated COST instrument itself, including difficulty affording care, reduced or quit work due to health, borrowing money or using savings for care, and treatment-adherence impact. The full COST instrument was not used because it was developed and validated for patients undergoing active cancer treatment, whereas this study involved a brief community-based survey of a nononcology veteran population. The 4 items were chosen to capture practical, patient-reported manifestations of health-related financial strain that may be relevant to future screening workflows—difficulty affording care, work disruption, borrowing or using savings, and treatment-adherence impact—while limiting respondent burden. These adapted items should not be interpreted as producing a validated COST score. Program use was assessed separately as reported use of available financial support resources. Thirteen participants did not answer the financial toxicity items; therefore, financial toxicity outcomes were available for 75 of 88 participants. The exact wording of all financial literacy and financial toxicity survey items, including response options and coding rules, is provided in Multimedia Appendix 1. Reporting details for the online survey component are provided in the completed CHERRIES (Checklist for Reporting Results of Internet E-Surveys) checklist (Checklist 1).

Demographic variables included age category, race and ethnicity, sex, education level, and marital status. Race and ethnicity was collected as a single self-reported category, with Hispanic analyzed as its own category. Sex was self-reported using male and female response options; gender identity was not separately assessed. Because the survey captured only binary self-reported sex, results involving this variable are reported in terms of sex, and gender identity–based interpretations are beyond the scope of these data. Race and sex were examined because prior veteran literature has documented disparities in financial capability and financial vulnerability across these groups. For subgroup analyses, race comparisons focused primarily on Black vs non-Black participants because of sample size constraints. The non-Black comparison group included participants who identified as White, Hispanic, Asian or Pacific Islander, other race and ethnicity, or preferred not to answer. This grouping was used only for exploratory analysis and should not be interpreted as implying that non-Black participants are a homogeneous group. Intersectional analyses were interpreted cautiously because subgroup counts were small. Health care setting and coverage variables, including Veterans Health Administration use, Medicare, Medicaid, private insurance, other insurance status, and payer source, were not assessed.

### Statistical Analysis

Descriptive statistics were used to summarize participant characteristics, financial literacy, and financial toxicity outcomes. Percentages were calculated using valid-response denominators. Group differences in categorical outcomes were evaluated using Fisher exact tests since cell sizes were small. All hypothesis tests were 2-sided. Given the exploratory nature of the analyses and the number of comparisons performed, *P* values are reported descriptively without correction for multiple testing; findings should not be interpreted as confirmatory.

Financial literacy was examined in two ways: (1) as a composite high-vs-low literacy variable and (2) at the item level for compound interest, inflation, and retirement strategy. Composite and item-level literacy differences were compared across sex and race subgroups. Due to small subgroup counts, Black female veterans were examined descriptively and inferentially only in limited exploratory comparisons.

Associations between financial literacy and each financial toxicity outcome were examined using unadjusted logistic regression in 2 specifications: binary financial literacy status (low vs high) and continuous financial literacy score (0‐3). Separate unadjusted logistic regression models were run for each financial toxicity outcome: treatment-adherence impact, reduced or quit work, borrowing money or using savings for care, and difficulty affording care. Odds ratios (ORs) in continuous models were interpreted per 1-point increase in financial literacy score. Because work disruption emerged as the clearest unadjusted signal, and because age and education were considered plausible confounders of both financial literacy and financial strain, we additionally conducted a minimally adjusted sensitivity analysis for the reduced-or-quit-work outcome controlling for age category and education level. Given the modest sample size and limited number of work-disruption events, age and education were modeled parsimoniously as ordered categories to reduce overfitting. All regression findings were interpreted as exploratory and hypothesis-generating.

Statistical code used for the analyses is available from the corresponding author on reasonable request.

### Ethical Considerations

The study was reviewed by the WCG Institutional Review Board and determined to be exempt (IRB 2155779) on November 27, 2024. The study was conducted in accordance with applicable ethical standards for human subjects research. Informed consent was obtained from all participants involved in the study through voluntary anonymous survey participation after the presentation of study information. Consistent with the exemption determination, formal written informed consent was not required; however, participants were informed of the study’s purpose, voluntary nature, and anonymity of their responses prior to completing the survey. No individual person’s data, images, or videos are included in this manuscript.

## Results

### Participant Characteristics

The final analytic sample included 88 US veterans, as summarized in Table 1. Of the 88 participants, the sample comprised 59 (67%) men and 29 (33%) women. By race and ethnicity, 51 (58%) participants identified as White, 24 (27%) as Black, 7 (8%) as Hispanic, and 4 (5%) as Asian or Pacific Islander; 2 (2%) participants identified as another category or preferred not to answer. Age was reported in categories, with the largest proportions in the 30 to 40 and 50 to 60 year groups. Most participants reported at least some college education (80/88, 91%). The majority were married or partnered (60/88, 68%), while 16 of 88 (18%) were single, and 12 of 88 (14%) were divorced or separated.

**Table 1.** Characteristics of included participants (N=88)^a^.

Characteristics	Participants, n (%)
Age (y)	
18‐30	7 (8)
30‐40	20 (23)
40‐50	16 (18)
50‐60	20 (23)
60‐70	14 (16)
≥70	11 (13)
Self-reported sex	
Male	59 (67)
Female	29 (33)
Race and ethnicity	
White	51 (58)
Black	24 (27)
Hispanic	7 (8)
Asian or Pacific Islander	4 (5)
Other	2 (2)
Education	
Graduate degree (masters or higher)	39 (44)
College or undergraduate	24 (27)
Some college	17 (19)
High school	8 (9)
Marital status	
Married or partnered	60 (68)
Single	16 (18)
Divorced or separated	12 (14)
Race-by-sex subgroups	
White	
Male	34 (39)
Female	17 (19)
Black	
Male	18 (21)
Female	6 (7)
Hispanic	
Male	4 (5)
Female	3 (3)
Asian or Pacific Islander	
Male	2 (2)
Female	2 (2)
Other	
Male	1 (1)
Female	1 (1)

a Percentages may not sum to 100% due to rounding.

### Financial Literacy by Race and Self-Reported Sex

Composite and item-level financial literacy findings are shown in Tables 2 and 3. In this exploratory sample, female veterans showed lower observed rates of high financial literacy than male veterans (15/29, 52% vs 48/59, 81%; Fisher exact test *P*=.006). At the item level, women also had lower correct-response rates for compound interest (10/29, 35% vs 36/59, 61%; *P*=.02) and inflation (14/29, 48% vs 43/59, 73%; *P*=.03), while retirement strategy did not differ at conventional thresholds by self-reported sex. All sex-based comparisons are similarly reported descriptively, given the exploratory design and the absence of correction for multiple testing.

**Table 2.** Composite high financial literacy by demographic subgroup^a^.

Comparison	Group 1, n/N (%)	Group 2, n/N (%)	Fisher exact test *P* value
Male vs female	Male: 48/59 (81)	Female: 15/29 (52)	.006
Black vs non-Black	Black: 15/24 (63)	Non-Black: 48/64 (75)	.29
Black women vs all others	Black female: 2/6 (33)	All others: 61/82 (74)	.05

a Fisher exact tests were used throughout. High financial literacy was defined as 2 to 3 correct responses out of 3 benchmark financial literacy items*.*

**Table 3.** Financial literacy by item-level correct responses.

Item	Black, n/N (%)	Non-Black, n/N (%)	Fisher exact test *P* value	Female, n/N (%)	Male, n/N (%)	Fisher exact test *P* value
Compound interest	10/24 (42)	36/64 (56)	.24	10/29 (34)	36/59 (61)	.02
Inflation	11/24 (46)	46/64 (72)	.03	14/29 (48)	43/59 (73)	.03
Retirement strategy	15/24 (63)	56/64 (88)	.01	21/29 (72)	50/59 (85)	.25

Black veterans had lower composite high-literacy rates than non-Black veterans (15/24, 63% vs 48/64, 75%), although this difference did not reach conventional thresholds (*P*=.29). At the item level, Black veterans had lower correct-response rates on inflation (11/24, 46% vs 46/64, 72%; *P*=.03) and retirement strategy (15/24, 63% vs 56/64, 88%; *P*=.01), while the difference in compound interest did not reach conventional thresholds (*P*=.24). All item-level comparisons are reported descriptively, given the exploratory design and absence of correction for multiple testing.

Among Black female veterans, 2 of 6 (33%) met the high-literacy threshold, compared with 61 of 82 (74%) among all other participants (Fisher exact test *P*=.052). This comparison is exploratory and descriptive only, given the small subgroup size. These findings are summarized in Tables 2 and 3.

### Financial Toxicity Indicators

Financial toxicity outcomes are summarized in Table 4 using valid-response denominators among the 75 participants who completed the financial toxicity items. Overall, 12 of 75 (16%) participants reported difficulty affording treatment or medications, 19 of 75 (25%) reported reduced or quit work because of health, 28 of 75 (37%) reported treatment-adherence impact, and 14 of 75 (19%) reported borrowing money or using savings for care. Financial support program use was reported by 22 of 75 (29%) participants.

**Table 4.** Financial toxicity indicators: overall sample (N=75 with complete data).

Indicator	n (%)^a^
Medical expenses <10% of income	52 (69)
Treatment adherence impact (any)	28 (37)
Reduced or quit work	19 (25)
Difficulty affording care	12 (16)
Borrowed or used savings for care	14 (19)
Financial support program use	22 (29)

a Denominators reflect valid responses among the 75 participants with complete financial toxicity data.

The work-disruption indicator may not have been applicable to all respondents, for example, those who were unemployed or already retired at the time of the survey, and the denominator therefore includes potentially nonapplicable participants which may affect prevalence estimates and should be considered when interpreting the work-disruption association.

### Association Between Financial Literacy and Financial Toxicity

Cross-tabulations and unadjusted logistic regression results are presented inTables 5-7. Veterans with low financial literacy (0‐1 correct) had numerically higher rates of all 4 financial toxicity outcomes than those with high financial literacy (2‐3 correct), although not all comparisons reached conventional thresholds. Treatment-adherence impact was reported by 9 of 22 (41%) participants with low financial literacy compared with 19 of 53 (36%) participants with high financial literacy. Reduced or quit work was reported by 9 of 22 (41%) participants with low financial literacy compared with 10 of 53 (19%) participants with high financial literacy. Borrowing money or using savings for care was reported by 6 of 22 (27%) participants with low financial literacy compared with 8 of 53 (15%) participants with high financial literacy. Difficulty affording care was reported by 5 of 22 (23%) participants with low financial literacy compared with 7 of 53 (13%) participants with high financial literacy.

**Table 5.** Financial toxicity indicators by financial literacy levels^a^.

Outcome	Low financial literacy, 0‐1 correct, n/N (%)	High financial literacy, 2‐3 correct, n/N (%)
Treatment-adherence impact	9/22 (41)	19/53 (36)
Reduced or quit work	9/22 (41)	10/53 (19)
Borrowed money or used savings for care	6/22 (27)	8/53 (15)
Difficulty affording care	5/22 (23)	7/53 (13)

a Low financial literacy was defined as 0-1 correct responses, and high financial literacy was defined as 2-3 correct responses.

**Table 6.** Financial toxicity indicators by unadjusted logistic regression results.

Outcome^a^	OR^b^ for low vs high financial literacy (95% CI; *P* value)	OR per 1-point increase in financial literacy score (95% CI; *P* value)
Treatment-adherence impact	1.24 (0.45‐3.43; *P*=.68)	0.82 (0.51‐1.32; *P*=.42)
Reduced or quit work	2.98 (1.00‐8.88; *P*=.051)	0.56 (0.33‐0.95; *P*=.03)
Borrowed money or used savings for care	2.11 (0.63‐7.02; *P*=.22)	0.59 (0.33‐1.05; *P*=.07)
Difficulty affording care	1.93 (0.54‐6.92; *P*=.31)	0.73 (0.40‐1.34; *P*=.32)

a Unadjusted logistic regression models were run separately for each financial toxicity outcome.

b OR: odds ratio.

**Table 7.** Financial toxicity indicators by minimally adjusted work-disruption sensitivity model^a^.

Predictor	Adjusted OR^b^ (95% CI)	*P* value
Financial literacy score, per 1-point increase	0.55 (0.28‐1.08)	.08
Age category	1.75 (1.09‐2.82)	.02
Education level	0.41 (0.22‐0.77)	.005

a Data show minimally adjusted sensitivity model for reduced or quit work, controlling for age category and education level. Age and education were modeled as ordered categories. N=75 reflects participants who completed the financial toxicity items.

b OR: odds ratio.

In unadjusted logistic models using low vs high financial literacy, lower financial literacy was directionally associated with higher odds of each financial toxicity outcome. The strongest unadjusted signal was observed for work disruption (OR 2.98, 95% CI 1.00‐8.88; *P*=.051), which did not reach conventional thresholds in the binary model. In continuous score models, each 1-point increase in financial literacy score was associated with lower odds of reduced or quit work (OR 0.56, 95% CI 0.33‐0.95; *P*=.03), though this finding should be interpreted descriptively given the exploratory design and absence of multiple testing correction. Associations for treatment-adherence impact, borrowing or savings use, and difficulty affording care were directionally similar but did not reach conventional thresholds. In a minimally adjusted sensitivity model controlling for age category and education level, the association between continuous financial literacy score and reduced or quit work remained directionally similar but was attenuated and did not reach conventional thresholds (adjusted OR 0.55, 95% CI 0.28‐1.08; *P*=.08). Within the same minimally adjusted model, higher education level was independently associated with lower odds of reduced or quit work (adjusted OR, 0.41; 95% CI, 0.22‐0.77; *P*=.005), whereas older age category was associated with higher odds of reduced or quit work (adjusted OR 1.75, 95% CI 1.09‐2.82; *P*=.02). The corresponding minimally adjusted model using dichotomous financial literacy status showed a similar pattern (adjusted OR for low vs high financial literacy, 2.90, 95% CI 0.71‐11.75; *P*=.13).

As a secondary sensitivity analysis, we examined an alternative high-literacy threshold consistent with prior NFCS-based research. Because high financial literacy is often defined as 3 of 3 correct responses in NFCS-based work, we conducted a sensitivity analysis using that stricter threshold. Results for work disruption were directionally similar, with higher odds among participants scoring 0 to 2 correct compared with those scoring 3 of 3 correct, but the association did not reach conventional thresholds (OR 2.26, 95% CI 0.72‐7.12; *P*=.17). In a minimally adjusted model controlling for age category and education level, the direction was unchanged but remained imprecise (adjusted OR 2.53, 95% CI 0.69‐9.32; *P*=.16).

### Subgroup Patterns in Financial Toxicity

Exploratory subgroup comparisons are presented in Table 8. In descriptive analyses, Black female veterans with available data had numerically higher reported financial toxicity across all 4 assessed financial toxicity outcomes. Among Black female veterans with available data, 3 of 5 (60%) reported difficulty affording care, 4 of 5 (80%) reported reduced or quit work, 3 of 5 (60%) reported borrowing money or using savings for care, and 5 of 5 (100%) reported treatment-adherence impact.

**Table 8.** Financial toxicity indicators by race and sex subgroup (valid-response denominators)^a^.

Subgroup	N (toxicity data)	Difficulty affording care, n/N (%)	Reduced work, n/N (%)	Borrowed or savings, n/N (%)	Adherence impact, n/N (%)	Program use, n/N (%)
White male	33	2/33 (6)	5/33 (15)	3/33 (9)	6/33 (18)	11/33 (33)
White female	17	3/17 (18)	4/17 (24)	4/17 (24)	9/17 (53)	5/17 (29)
Black male	7	3/7 (43)	5/7 (71)	1/7 (14)	4/7 (57)	3/7 (43)
Black female	5	3/5 (60)	4/5 (80)	3/5 (60)	5/5 (100)	0/5 (0)
Hispanic male	4	1/4 (25)	1/4 (25)	2/4 (50)	3/4 (75)	1/4 (25)
Hispanic female	3	0/3 (0)	0/3 (0)	0/3 (0)	1/3 (33)	0/3 (0)
Asian male	2	0/2 (0)	0/2 (0)	0/2 (0)	0/2 (0)	0/2 (0)
Asian female	2	0/2 (0)	0/2 (0)	0/2 (0)	0/2 (0)	0/2 (0)
Other female	1	0/1 (0)	1/1 (100)	1/1 (100)	0/1 (0)	0/1 (0)

a Percentages are based on item-specific valid-response denominators. All subgroup counts were small; all results are descriptive only and should not be interpreted as confirmatory. For reference, Fisher exact comparisons of Black female veterans vs all other participants yielded *P*=.04 for difficulty affording care, *P*=.01 for reduced work, *P*=.04 for borrowed money or use of savings, and *P*=.006 for treatment-adherence impact; given n=5, these values are not reliable for inference. Program use did not differ at conventional thresholds (*P*=.31).

In a descriptive comparison limited to female veterans with available financial toxicity data, Black female veterans also had numerically higher rates of all 4 assessed financial toxicity outcomes than other female veterans. Difficulty affording care was reported by 3 of 5 (60%) Black female veterans compared with 3 of 23 (13%) other female veterans, reduced or quit work by 4 of 5 (80%) vs 5 of 23 (22%), borrowing money or using savings for care by 3 of 5 (60%) vs 5 of 23 (22%), and treatment-adherence impact by 5 of 5 (100%) vs 10 of 23 (43%). Financial support program use was reported by 0 of 5 (0%) Black female veterans compared with 5 of 23 (22%) other female veterans. These comparisons are descriptive only due to the very small number of Black female veterans with complete financial toxicity data.

Given the very small cell size (n=5), these comparisons are descriptive only, and no inferential conclusions should be drawn. Fisher exact test *P* values are reported in the table footnote for completeness but should not be interpreted as statistically reliable. Financial support program use was zero among Black female veterans with available data.

Among White veterans, women reported numerically higher rates than men for 4 of 5 financial toxicity indicators, although subgroup counts were small, and these comparisons were descriptive only. Financial support program use was lower among White female veterans than among White male veterans.

## Discussion

### Principal Findings

In this exploratory survey of 88 US veterans, 4 principal findings emerged. First, female veterans had lower financial literacy than male veterans, most notably regarding compound interest and inflation. Second, Black veterans showed lower correct-response rates than non-Black veterans for inflation and retirement strategy, although composite high-literacy rates did not differ significantly by race. Third, lower financial literacy was directionally associated with higher financial toxicity across all 4 assessed outcomes in unadjusted analyses, with the clearest signal observed for work disruption; however, the work-disruption association was attenuated and no longer reached conventional thresholds after minimal adjustment for age and education. Fourth, financial literacy gaps and financial toxicity burden appeared to follow partially distinct demographic patterns, with the numerically highest reported toxicity burden observed among Black female veterans in this small exploratory subgroup.

Female veterans demonstrated lower financial literacy than their male counterparts on both composite and item-level measures, with differences most pronounced in compound interest and inflation knowledge. These findings are consistent with prior work documenting sex-based differences in financial capability among veterans [8,9] and with broader literature on sex-based differences in financial literacy [14-16]. This study builds on prior work by suggesting specific concept domains that contributed to the observed differences rather than examining financial literacy as a single aggregate score. Compound interest and inflation knowledge may be especially relevant to health-related financial decision-making, as inflation affects the real value of fixed incomes and veterans’ benefits over time, while compound interest affects the long-term cost of borrowing for medical care. Retirement strategy knowledge may similarly affect long-term financial resilience and the capacity to absorb health care–related income shocks without depleting savings.

Black veterans showed lower correct-response rates than non-Black veterans specifically for inflation and retirement strategy, although overall composite high-literacy rates did not differ at conventional thresholds by race. It should be noted that observed item-level differences between Black and non-Black veterans were not adjusted for educational attainment or age composition, and it remains possible that these factors partially account for the observed patterns. The item-level approach offers more granular insight than composite measures and may help identify potential targets for future education or navigation efforts that may not be apparent from aggregate scores alone.

In unadjusted logistic models, lower financial literacy was directionally associated with higher odds of all 4 assessed financial toxicity outcomes. The clearest unadjusted association was observed for work disruption, where each 1-point increase in financial literacy score was associated with lower odds of reducing or quitting work. However, in a minimally adjusted sensitivity model controlling for age category and education level, this association was attenuated and no longer reached conventional thresholds. The other 3 outcomes showed similar directional patterns in unadjusted analyses but did not reach conventional thresholds. Taken together, these findings suggest a possible relationship between lower financial literacy and health-related financial strain, but they do not establish an independent association in this sample. The observed attenuation after adjustment and the small sample size underscore the need for larger, adequately powered studies with multivariable modeling. Furthermore, the sample’s high educational attainment (91% with at least some college) is not representative of the broader veteran population and may have influenced estimates of financial strain, meaning prevalence estimates in broader veteran populations may differ from those observed here. Separately, financial toxicity indicators were observed even though many participants reported medical expenses below 10% of income. This pattern is consistent with the possibility that health-related financial strain may reflect not only direct out-of-pocket spending but also broader pressures related to employment disruption, savings depletion, and care access.

Finally, financial literacy gaps and financial toxicity burdens appeared to follow partially different demographic patterns in this sample. For financial literacy, the clearest composite difference was observed by sex, with female veterans demonstrating lower high-literacy rates than male veterans. In contrast, the highest financial toxicity burden was observed among Black female veterans, who comprised a small exploratory subgroup. Among Black female veterans with available data, elevated rates were observed across all 4 assessed financial toxicity outcomes. This pattern should not be interpreted as evidence that all female veterans had uniformly higher financial toxicity than male veterans. Rather, it suggests that financial literacy gaps and financial toxicity may reflect overlapping but distinct sources of vulnerability. Financial literacy interventions targeting female veterans may address some knowledge gaps but may not fully address the structural, economic, health care access, and benefit-navigation factors that could contribute to financial toxicity among Black female veterans. These findings are preliminary and require confirmation in adequately powered intersectional analyses.

### Comparison With Prior Work

These findings are broadly consistent with, but extend, prior national-level work by Mottola and Skimmyhorn [8] and Skimmyhorn et al [9], which documented financial capability disparities by race and sex among veterans. Furthermore, this study adds exploratory evidence in 3 areas not captured in those reports: concept-level assessment of specific financial literacy gaps, preliminary examination of the association between financial literacy and health-related financial toxicity, and descriptive documentation of financial toxicity patterns, including treatment-adherence impact and work disruption, in a veteran population outside of a clinical disease context.

Beyond the veteran context, these findings highlight a broader public health concern: financial strain related to health care does not arise solely from medical need but also from the interaction of economic resources, financial knowledge, and access to supportive systems. In that sense, the present results may have relevance for other vulnerable populations facing cost-related barriers to care, employment disruption, or difficulty navigating complex benefit structures [20-22]. The concept-level approach used here may therefore be useful not only for veteran populations but also for identifying actionable financial literacy gaps in other groups at risk of health-related financial strain [14-16].

From a public health and policy perspective, these findings suggest potential value in integrating brief financial toxicity screening into veteran care workflows, particularly for indicators such as work disruption and treatment-adherence strain. Targeted financial literacy education addressing core concepts, particularly compound interest, inflation, and retirement strategy, may be more focused than generalized financial education and may warrant further evaluation, particularly among female veterans. Such efforts may be strengthened with active benefit-navigation support, as financial support program utilization was low (29%) and did not differ significantly even among the subgroup with the highest reported toxicity burden.

### Public Health Informatics Implications

These findings may inform future evaluations of brief informatics-enabled financial toxicity screening workflows for veterans, with particular attention to work disruption, borrowing money or using savings for care, difficulty affording care, treatment-adherence impact, and low financial support program utilization. However, implementation would likely differ across care environments. In the Veterans Health Administration or other integrated systems, these domains could potentially be incorporated into electronic health record–based screening tools, patient portal questionnaires, or referral workflows. In community-based, private, Medicare, Medicaid, or uninsured or underinsured care settings, similar screening may require lower-resource approaches, such as brief paper or digital questionnaires, community organization referral pathways, social work referral protocols, or partnerships with veteran service organizations. Because this study did not assess Veterans Health Administration use, insurance type, payer source, or care coordination setting, we could not evaluate how the health care environment influenced financial toxicity or the feasibility of screening implementation. Future research should compare screening, referral, and benefit-navigation approaches across Veterans Health Administration and community-based care settings and assess whether these workflows improve identification, referral completion, treatment adherence, and downstream financial and health outcomes.

### Strengths and Limitations

This study has several notable strengths. It uses standardized NFCS benchmark items to assess concept-level financial literacy, enabling comparison with prior national-level research. By analyzing financial literacy at the concept level rather than only as a composite score, the study suggests specific knowledge domains—particularly compound interest, inflation, and retirement strategy—that may warrant further evaluation in future education or navigation interventions. The simultaneous examination of financial literacy and health-related financial toxicity within the same veteran sample addresses an important gap in the existing literature, which has generally examined these constructs separately. In addition, applying a COST-aligned framework to a nononcology veteran population extends this line of inquiry beyond its usual clinical context and captures patient-reported outcomes, including treatment-adherence impact and work disruption, that have not been extensively assessed in prior veteran financial capability research.

Several limitations should be acknowledged. First, the modest sample size (N=88; n=75 for financial toxicity analyses) limited statistical power, especially for subgroup analyses and multivariable modeling. Race-by-sex subgroups were particularly small, including Black female veterans (n=6) with complete financial toxicity data (n=5), and those findings should therefore be interpreted cautiously. Second, the study used a convenience sample that was also highly educated, with 91% (n=80) of participants reporting at least some college and 44% (n=39) reporting a graduate degree. This educational profile is not representative of the broader US veteran population, limits external validity, and may have influenced estimates of financial strain in this sample. Participant ZIP code, Area Deprivation Index, Social Vulnerability Index, Veterans Health Administration use, insurance type, payer source, and care coordination setting were not collected; therefore, we could not evaluate whether neighborhood socioeconomic context, health care coverage setting, or care environment influenced financial toxicity, financial support use, or the feasibility of screening and navigation workflows. Third, although a minimally adjusted sensitivity model was conducted for work disruption, residual confounding by income, marital status, employment context, and other unmeasured factors remains possible. Fourth, the cross-sectional design precludes causal inference and leaves open the possibility of reverse causation. Fifth, financial toxicity was assessed using a framework-aligned adaptation rather than the fully validated COST instrument. Sixth, findings involving dichotomized financial literacy were sensitive to threshold choice; when high financial literacy was defined more strictly as 3 of 3 correct responses, the direction of association with work disruption was similar, but estimates were attenuated and remained imprecise. The pragmatic 0‐1 vs 2‐3 threshold used in primary analyses is not the most common cut point in NFCS literature, and results should be interpreted with this in mind.

Several individual-level factors known to shape financial literacy and financial decision-making in aging populations were not assessed. We did not measure cognitive performance, frailty, neurobiological correlates, functional status, or financial exploitation vulnerability, each of which may be relevant to financial literacy and financial vulnerability in older adults. Lower performance on benchmark financial literacy concepts has been associated with cognitive impairment and structural brain differences in older adults [23], and frailty has been proposed as a correlate of perceived financial exploitation in older women [24]. Because a substantial proportion of this sample was older, these unmeasured factors may have contributed to the observed financial literacy and financial toxicity patterns, and we did not examine whether sex-based differences in financial literacy varied by age. Relatedly, heightened financial vulnerability documented among older Black women in other work [25] reinforces the value of examining intersectional subgroups with richer individual-level data than were available here. Finally, the 3 financial literacy items capture widely used benchmark concepts but do not cover all domains that may influence financial toxicity, such as debt literacy, insurance literacy, budgeting, credit use, benefit navigation, and health care cost understanding. Because participants were recruited within a US veteran context, findings should be generalized cautiously to non-US populations and health care systems with different financing structures.

### Conclusions

This exploratory study observed subgroup differences in specific financial literacy concepts among US veterans. Female veterans showed lower composite and item-level financial literacy than male veterans, with lower correct-response rates most evident for compound interest and inflation. Black veterans showed lower correct-response rates on inflation and retirement strategy than non-Black veterans, although composite high-literacy rates did not differ significantly by race. In unadjusted analyses, lower financial literacy was directionally associated with higher reported financial toxicity across all 4 assessed outcomes, with the clearest signal observed for work disruption; however, this association was attenuated after minimal adjustment for age and education. Black female veterans reported numerically elevated financial toxicity across all 4 assessed outcomes in a very small subgroup, and these findings should be interpreted cautiously.

Beyond describing exploratory subgroup patterns, this study highlights specific candidate data elements that could support future public health informatics approaches to detecting financial toxicity in veterans and other populations facing health care affordability challenges. Concept-level financial literacy gaps, work disruption, treatment-adherence impact, and low use of support programs may warrant evaluation as brief digital screening indicators and subgroup-aware risk-stratification variables. Future studies should evaluate whether integrating these indicators into electronic health record–based screening workflows, patient portal questionnaires, referral dashboards, and community-facing paper or digital referral tools could help clinicians and health systems identify financially vulnerable patients earlier and connect them to appropriate navigation and support services. Future research should also test whether such informatics-enabled strategies improve identification, referral completion, treatment adherence, and downstream financial and health-related outcomes in larger veteran cohorts and, where relevant, in other high-risk groups.

## Supplementary material

10.2196/97291 Multimedia Appendix 1 Survey items, response options, and coding rules used to assess financial literacy and financial toxicity.

10.2196/97291 Checklist 1 CHERRIES checklist.

## Abbreviations

CHERRIES: Checklist for Reporting Results of Internet E-Surveys
COST: Comprehensive Score for Financial Toxicity
NFCS: National Financial Capability Study
OR: odds ratio
STROBE: Strengthening the Reporting of Observational Studies in Epidemiology
