# Supplementary material for: Financial Literacy and Financial Toxicity Among US Veterans: Cross-Sectional Survey Informing Public Health Informatics Screening
Source: Online J Public Health Inform. 2026 Aug 7;18:e97291. doi: 10.2196/97291 (PMC13450884; doi:10.2196/97291)
Supplement: Supplementary file 1 [file ojphi-v18-e97291-s001.docx]

**Multimedia Appendix 1. Survey Items, Response Options, and Coding Rules Used to Assess Financial Literacy and Financial Toxicity**

**A. Financial literacy items used in primary analyses**

| **Domain** | **Exact survey item wording** | **Response options** | **Coding used in analyses** |
| --- | --- | --- | --- |
| Compound interest | **7. If you have $1,000 in a savings account earning 3% interest per year, how much will you have in the account at the end of 5 years?** | More than $1,150; Exactly $1,150; Less than $1,150; Don’t know | Correct = “More than $1,150”; all other responses, including “Don’t know” and nonresponse, coded as incorrect |
| Inflation | **8. Suppose the interest rate on your savings account is 2% per year and inflation is 3% per year. After one year, will the money in the account buy more than it does today, exactly the same, or less than today?** | More; The same; Less; Don’t know | Correct = “Less”; all other responses, including “Don’t know” and nonresponse, coded as incorrect |
| Retirement diversification | **9. Which of the following is typically the best strategy for long-term retirement savings?** | Investing in a mix of stocks and bonds; Investing in a single company’s stock; Keeping all your money in a savings account; Don’t know | Correct = “Investing in a mix of stocks and bonds”; all other responses, including “Don’t know” and nonresponse, coded as incorrect |

**Derived financial literacy variables**

**Composite financial literacy score:** sum of the 3 benchmark items above, range **0–3**

**Primary dichotomous literacy variable:** low literacy = **0–1** correct; high literacy = **2–3** correct

**Sensitivity dichotomous literacy variable:** lower literacy = **0–2** correct; high literacy = **3 of 3** correct

**B. Financial-toxicity-related items reported in the main manuscript**

| **Domain** | **Exact survey item wording** | **Response options** | **Coding used in analyses** |
| --- | --- | --- | --- |
| Medical expense share | **24. In the past 12 months, how much of your income has gone toward medical expenses (including treatments, medications, and co-pays)?** | Less than 10%; 10–25%; 26–50%; More than 50%; Not sure | Reported descriptively in Table 3 |
| Difficulty affording care | **25 b. Have you experienced difficulty affording treatments or medications prescribed for your condition?** | Yes; No | Binary outcome: Yes vs No |
| Work disruption | **27. Since being diagnosed, have you had to reduce work hours, retire early, or quit a job due to your condition?** | Yes; No | Binary outcome: Yes vs No |
| Borrowing / savings depletion | **29. Have you borrowed money, used credit cards, or depleted savings to pay for medical expenses in the past year?** | Yes; No | Binary outcome: Yes vs No |
| Treatment-adherence impact | **31. Do you feel your financial situation affects your ability to prioritize medical care or adhere to treatment plans?** | No; Yes, somewhat; Yes, significantly | Binary outcome: any adherence impact (“Yes, somewhat” or “Yes, significantly”) vs No |
| Financial support program use | **32. Have you accessed any financial support programs or benefits to help with the financial burden of your condition?** | Yes; No | Reported descriptively as support-program use |

**C. Additional financial-toxicity-related items collected but not included in the primary analyses reported in the main manuscript**

| **Domain** | **Exact survey item wording** | **Response options** | **Analytic use** |
| --- | --- | --- | --- |
| **Financial stability impact** | **25 a. On a scale of 1–5 (1 = Not at all, 5 = Extremely), how much have medical expenses related to your condition impacted your overall financial stability?** | **1–5 numeric scale** | **Collected, not included in primary analyses reported in the main manuscript** |
| **Ability to work / maintain income** | **26. Has your medical condition affected your ability to work or maintain consistent income?** | **No; Yes, somewhat; Yes, significantly** | **Collected, not included in primary analyses reported in the main manuscript** |
| **Financial stress managing medical needs** | **28. On a scale of 1–5, how much stress does your current financial situation cause you in managing your medical needs?** | **1–5 numeric scale** | **Collected, not included in primary analyses reported in the main manuscript** |
| **Confidence covering unexpected medical expenses** | **30. How confident are you in your ability to cover unexpected medical expenses in the future?** | **1–5 numeric scale** | **Collected, not included in primary analyses reported in the main manuscript** |

**D. Notes on coding**

1. Financial literacy items were adapted from benchmark items used in the National Financial Capability Study.
2. “Don’t know” responses and nonresponse were coded as incorrect for the 3 literacy items.
3. Financial toxicity items were framework-aligned with domains reflected in the Comprehensive Score for Financial Toxicity, but did **not** constitute the validated COST instrument itself.
4. Percentages in the main manuscript were calculated using valid-response denominators.
5. The work-disruption item may not have been applicable to all respondents, such as those unemployed or already retired at the time of survey completion.
