# Supplementary material for: Financial Literacy and Financial Toxicity Among US Veterans: Cross-Sectional Survey Informing Public Health Informatics Screening
Source: Online J Public Health Inform. 2026 Aug 7;18:e97291. doi: 10.2196/97291 (PMC13450884; doi:10.2196/97291)
Supplement: Supplementary file 2 [file ojphi-v18-e97291-s002.docx]

**Multimedia Appendix 2. CHERRIES Checklist**

*Checklist for Reporting Results of Internet E-Surveys (CHERRIES), completed for: “Financial Literacy and Financial Toxicity Among United States Veterans: A Preliminary Cross-Sectional Survey Informing Public Health Informatics Screening.”*

“Reported” = the item is already covered in the manuscript at the section noted. “Action needed” = a brief addition to the Methods (or this checklist) is recommended before resubmission. Reference: Eysenbach G. Improving the quality of web surveys: the Checklist for Reporting Results of Internet E-Surveys (CHERRIES). J Med Internet Res. 2004;6(3):e34.

| **Item** | **CHERRIES checklist question** | **Status and where addressed / action** |
| --- | --- | --- |
| **1. Design** | | |
| **Describe survey design** | Describe target population, sample frame. Is the sample a convenience sample or a defined population? | **Reported.** Anonymous, voluntary, self-administered cross-sectional survey of U.S. veterans (web link plus in-person). Non-probability convenience sample, explicitly stated as not nationally representative. See Methods, “Study Design and Population.” |
| **2. IRB approval and informed consent process** | | |
| **IRB approval** | Mention whether the study has been approved by an Institutional Review Board. | **Reported.** Reviewed by the WCG Institutional Review Board; determined exempt (IRB #2155779), Nov 27, 2024. See “Institutional Review Board Statement.” |
| **Informed consent** | Describe the informed consent process. Where were participants told the length of time, data storage, and investigator? | **Reported.** Participants were informed of the study’s purpose, voluntary nature, and anonymity prior to completing the survey; written consent waived under the exemption. See “Informed Consent Statement.” |
| **Data protection** | If any personal information was collected, state what mechanisms were used to protect unauthorized access. | **Reported.** Responses were collected anonymously through Google Forms and stored on a password-protected, access-restricted device and file accessible only to the study team; no personally identifying information was collected, and only de-identified data were deposited in figshare. |
| **3. Development and pre-testing** | | |
| **Development and testing** | State how the survey was developed, including whether usability and technical functionality were tested. | The survey items were adapted from established instruments (the NFCS 'Big Three' financial-literacy questions and domains from the COST framework); full item wording is provided in Multimedia Appendix 1. No formal pilot, usability, or technical pre-testing of the questionnaire was conducted prior to fielding |
| **4. Recruitment process and description of the sample** | | |
| **Open vs closed survey** | Was the survey open to any visitor, or was access restricted (e.g., to a panel)? | **Reported.** Open survey — distributed via an anonymous link with no login or registration. |
| **Contact mode** | Indicate how the sample was contacted (email, offline, web-page postings, etc.). | **Reported.** Veteran-focused community contacts, online veteran networks, and in-person outreach at local community events. See Methods, “Study Design and Population.” |
| **Advertising the survey** | How/where was the survey announced or advertised? | **Reported.** Through veteran community contacts/networks and community events; in-person outreach primarily in the Northern Virginia/Washington, DC area. |
| **5. Survey administration** | | |
| **Web / email** | State whether it was a web-based or email survey. | **Reported.** Web-based anonymous link plus in-person self-administration. Reported in Methods. |
| **Context** | Describe the website/context in which the survey was presented. | **Reported.** Veteran community settings and online veteran networks. Reported in Methods. |
| **Mandatory / voluntary** | Was the survey mandatory or voluntary? | **Reported.** Voluntary. Reported in Methods and Informed Consent Statement. |
| **Incentives** | Were incentives offered? | **Reported.** None — “No financial incentive was provided.” Reported in Methods. |
| **Time / date** | In what time frame were the data collected? | **Reported.** July 2024 to April 2025. Reported in Methods. |
| **Randomization / adaptive questioning** | Were items or pages randomized? Were adaptive questions used to reduce items? | Survey items were presented in a fixed order, with no item/page randomization and no adaptive questioning |
| **Number of items / screens** | What was the number of questionnaire items per page and the number of pages? | The survey was administered as a single Google Forms questionnaire and included demographic items, three financial-literacy items, and financial-toxicity-related items. Full item wording and analytic coding are provided in Multimedia Appendix 1. |
| **Completeness check / review step** | Were completeness checks done before submission? Could respondents review and change answers? | Completeness was partially enforced: the first 23 items (covering demographics and financial literacy) were marked as required, while the remaining items (financial-toxicity questions) were optional. This is consistent with the observed missingness, as financial-toxicity items were left unanswered by some respondents. Google Forms' back/review functionality allowed respondents to review and change answers before submission |
| **6. Response rates** | | |
| **Unique site visitor / view rate** | How were repeated visitors counted; what was the view rate? | **Reported.** Could not be determined because recruitment was anonymous and community-based; explicitly acknowledged in Methods |
| **Participation rate** | Ratio of unique visitors who agreed to participate to first survey-page visitors. | **Reported.** Not determinable for the same reason (anonymous, no denominator). Acknowledged in Methods and Limitations. |
| **Completion rate** | Ratio of users who finished the survey to those who agreed to participate. | **Reported.** A formal completion rate could not be calculated because the number of individuals who viewed or began the anonymous survey was not known. Among submitted responses included in the analysis, 75 of 88 participants (85%) completed the financial-toxicity items; the financial-literacy analytic sample was 88. |
| **7. Preventing multiple entries from the same individual** | | |
| **Cookies / IP / log-file checks** | Were cookies, IP address checks, or log-file analysis used to prevent duplicate entries? | No technical measures to prevent or detect duplicate submissions (e.g., cookies, IP-address checks, or log-file analysis) were used. Because the survey was anonymous and collected no identifying information, the possibility of multiple submissions by the same individual cannot be excluded. |
| **Registration** | If a registration/log-in was used, describe it. | **Reported.** The survey used an open, anonymous link with no registration or log-in |
| **8. Analysis** | | |
| **Handling of incomplete questionnaires** | Were incomplete questionnaires analyzed? Was a cut-off defined? | **Reported.** Participants missing all three literacy items were excluded from literacy analyses; toxicity analyses were limited to the 75 participants with complete toxicity data; valid-response denominators were used throughout. See “Study Design and Population” and “Statistical Analysis.” |
| **Atypical timestamps** | Were submissions with atypical completion times excluded? | Response timestamps were not used to screen or exclude submissions; no responses were removed on the basis of completion time |
| **Statistical correction** | Was any weighting or statistical correction used (e.g., for non-representativeness)? | **Reported.** No weighting was applied; analyses were descriptive/exploratory and P values were reported without multiple-comparison correction, as stated in “Statistical Analysis.” |
